# Trimethylation of Histone H3 Lysine 36 by Human Methyltransferase PRDM9 Protein*

**DOI:** 10.1074/jbc.M113.523183

**Published:** 2014-03-14

**Authors:** Mohammad S. Eram, Susan P. Bustos, Evelyne Lima-Fernandes, Alena Siarheyeva, Guillermo Senisterra, Taraneh Hajian, Irene Chau, Shili Duan, Hong Wu, Ludmila Dombrovski, Matthieu Schapira, Cheryl H. Arrowsmith, Masoud Vedadi

**Affiliations:** From the ‡ Structural Genomics Consortium, University of Toronto, Toronto, Ontario M5G 1L7,; the ¶ Department of Pharmacology and Toxicology, University of Toronto, Toronto, Ontario M5S 1A8, and; the § Ontario Cancer Institute and Department of Medical Biophysics, University of Toronto, Toronto, Ontario M5G 2M9, Canada

**Keywords:** Cancer, Enzyme Inhibitors, Enzyme Kinetics, Epigenetics, Histone Methylation, Methyltransferase

## Abstract

PRDM9 (PR domain-containing protein 9) is a meiosis-specific protein that trimethylates H3K4 and controls the activation of recombination hot spots. It is an essential enzyme in the progression of early meiotic prophase. Disruption of the *PRDM9* gene results in sterility in mice. In human, several PRDM9 SNPs have been implicated in sterility as well. Here we report on kinetic studies of H3K4 methylation by PRDM9 *in vitro* indicating that PRDM9 is a highly active histone methyltransferase catalyzing mono-, di-, and trimethylation of the H3K4 mark. Screening for other potential histone marks, we identified H3K36 as a second histone residue that could also be mono-, di-, and trimethylated by PRDM9 as efficiently as H3K4. Overexpression of PRDM9 in HEK293 cells also resulted in a significant increase in trimethylated H3K36 and H3K4 further confirming our *in vitro* observations. Our findings indicate that PRDM9 may play critical roles through H3K36 trimethylation in cells.

## Teaser

**Background:** Histone methyltransferase PRDM9 marks recombination hot spots and is implicated in sterility and cancers.

**Results:** We identified histone H3K36 as a new methylation mark for PRDM9.

**Conclusion:** PRDM9 is a highly active histone methyltransferase that trimethylates H3K4 and H3K36.

**Significance:** Discovering PRDM9 as a H3K36 methyltransferase could lead to identifying the roles that PRDM9 may play in human fertility and susceptibility to cancer.

## Introduction

Methylation of histones is a post-translational modification involved in the regulation of gene expression (1). Protein methyltransferases are enzymes that transfer a methyl group from the donor *S*-adenosylmethionine (AdoMet)^4^ to lysine (protein-arginine methyltransferases) (2) or arginine (protein-arginine methyltransferases) (3) residues. Lysine residues can accept up to three methyl groups on their ϵ-group (2, 4, 5), whereas arginine residues can accept up to two methyl groups on their guanidinyl group (3, 6). The most common sites of histone lysine methylation are histone H3 lysine 4 (H3K4), H3K9, H3K27, H3K36, H3K79, and histone H4 lysine 20 (H4K20) (7).

There are significant differences in specificity of protein-arginine methyltransferases for specific lysine residues on different histones as well as their ability to mono-, di-, or trimethylate the target residues (8). For example, SETD7 (SET7/9) only monomethylates H3K4 (9), mixed lineage leukemia protein-1 (MLL1) mostly mono- and dimethylates H3K4 (10), NSD1, NSD2, and NSD3, three members of the nuclear receptor SET domain-containing (NSD) family of proteins mono- and dimethylate lysine 36 of histone 3 (H3K36) (11, 12), and SETD2 trimethylates H3K36 (13). Abnormal changes in the methylation state of histone marks have been widely implicated in many diseases including cancers (14–16).

The PRDM (PR domain-containing) protein family derives its name from sharing the N-terminal PR domain originally identified in PRDM1 (positive regulatory domain 1-binding factor 1; PRDI-BF1) and PRDM2 (retinoblastoma-interacting zinc finger protein 1; RIZ1) (17, 18). The PR domain is related to the SET (methyltransferase) domain of protein-arginine methyltransferases (19). The two domains share an amino acid sequence identity of 20–30% and the PR domain is thought to be derived evolutionarily from the SET domain (20). There are 17 PRDM protein orthologs in primates and all but PRDM11 contain a variable number of C2H2 zinc finger repeats (21). The zinc finger domains allow PRDM proteins to bind directly to DNA in a sequence-specific manner (22). Although the SET catalytic (H/R)*XX*(NH)*X*C motif (23) is absent in PRDM proteins, methyltransferase activity has been detected for the PR domains of PRDM2, PRDM3, PRDM6, PRDM8, PRDM9, and PRDM16 (24–29). Some PRDMs act as tumor suppressors (*e.g.* PRDM1, -2, -5, and -12) and others are oncogenic (*e.g.* PRDM3, -13, -14, and -16) (reviewed by Hohenauer and Moore (22)).

PRDM9 is the only member of the PR domain family whose expression is restricted to germ cells entering meiotic prophase (28). In addition to its PR domain, the protein contains a Krüppel-associated box domain (30) and anywhere from 8 to 18 C2H2 zinc finger repeats (31). The zinc finger domain of the main PRDM9 allele has been shown to bind to a 13-mer recombination hot spot motif on DNA (32, 33). Hot spot motifs are sites of DNA double strand breaks localized to 1–2-kb regions of the genome where homologous recombination takes place during meiosis (34). Trimethylation of H3K4 is a mark for the initiation of recombination in yeast and mice (35–37). PRDM9 has been shown to catalyze the trimethylation of H3K4 both *in vitro* and *in vivo* (28) and is the only locus known to specify hot spots in humans (38). The mechanism of this hot spot activator is proposed to begin with DNA binding via zinc finger domain, trimethylation of H3K4, followed by the initiation of double strand breaks by the topoisomerase-like protein SPO11 (39).

Targeted disruption of PRDM9 in mice causes sterility in both sexes as a result of impaired double strand break repair, deficient pairing of homologous chromosomes, and deficient sex body formation (28). In human, two SNPs, C614T in exon 6 and T1086C in exon 9, in PRDM9 were found to occur more frequently in Japanese patients with azoospermia caused by meiotic arrest than in the healthy control group (40). However, there was no difference detected in the methylation activity *in vitro* between normal PRDM9 and T1086C PRDM9, which caused a Tyr to His missense mutation. Three additional SNPs were found in another group of sterile male patients: two exonic SNPs, G17353T (G433V) and C18109G (T685R), and an intronic SNP, G15549T (41). These SNPs were identified in patients with azoospermia, but not in fertile subjects. PRDM9 is also recurrently mutated (11%) in head and neck squamous cell carcinoma (42).

*PRDM9* has been identified as a meiosis-specific cancer/testis gene (43). These genes encode cancer/testis antigens that are a group of cancer-specific biomarkers expressed in the testes of healthy adults that can also be activated in cancers. PRDM9 protein has been detected in the human testicular embryonic carcinoma cell line NTERA-2 and could potentially be used as an antigenic target in clinical applications. Another study linking PRDM9 with cancer found that an excess of rare *PRDM9* alleles were found in children affected by B-cell precursor acute lymphoblastic leukemia (B-ALL) and their parents (44).

Here we report on substrate specificity and kinetic characterization of PRDM9. Our results indicate that PRDM9 is a highly active histone methyltransferase that mono-, di-, and trimethylates H3K4, consistent with previous reports. We also report that H3K36 is a novel substrate for PRDM9.

## EXPERIMENTAL PROCEDURES

### 

#### 

##### Expression and Purification of PRDM9

The wild-type *PRDM9* gene (amino acids 195–385) was amplified by PCR and subcloned into the pET28a-MHL vector (GenBank^TM^ EF456735). The protein was overexpressed in *E. coli* BL21(DE3)pRARE2-V2R cells (SGC Toronto) by the addition of 1 mm isopropyl 1-thio-d-galactopyranoside and incubated overnight at 15 °C. Harvested cells were re-suspended in 20 mm Tris-HCl buffer, pH 7.5, with 500 mm NaCl, 5 mm imidazole, and 5% glycerol and flash-frozen in the presence of protease inhibitor (0.1 mm PMSF). The cells were thawed and lysed chemically with 0.5% CHAPS in the presence of 3 mm 2-mercaptoethanol followed by sonication for 10 min on ice at a frequency of 8.5 with 10 s on and off. The crude extract was clarified by high-speed centrifugation (16,000 × *g* for 1 h). The cleared lysate was loaded onto a Hi-Trap, 5-ml Ni-Chelating HP column using the AKTA system. The column was washed with wash buffer (20 mm Tris-HCl, pH 7.5, 500 mm NaCl, 5% glycerol, and 30 mm imidazole) and the His-tagged PRDM9 protein was eluted in 20 mm Tris-HCl buffer, pH 7.5, 500 mm NaCl, 5% glycerol, and 250 mm imidazole.

Following addition of 3 mm 2-mercaptoethanol, the eluate was loaded onto a Superdex 200 26/60 column equilibrated with 20 mm Tris-HCl, pH 7.5, and 150 mm NaCl. The His tag was cleaved overnight at 4 °C with tobacco etch virus protease. The NaCl in the buffer containing cut protein was diluted to 50 mm prior to injection onto a Source30Q (GE Healthcare Life Sciences) anion exchange column, which was pre-equilibrated with 3 column volumes of 20 mm Tris-HCl buffer, pH 8.0. The protein was eluted by addition of a salt gradient created by 3 column volumes of 20 mm Tris-HCl buffer, pH 8.0, containing 1 m NaCl and 20 mm Tris-HCl buffer, pH 8.0. The pure PRDM9 eluted at 150 and 250 mm NaCl. Fractions with higher than 95% purity based on SDS-PAGE and Coomassie Blue staining were pooled, followed by addition of 2-mercaptoethanol to 3 mm. Pooled fractions were concentrated, flash-frozen, and stored at −80 °C.

##### Nucleosome Preparation

Recombinant nucleosome with wild-type histone H3 (WT) and truncated histone H3 (ΔH3; missing the first 11 residues) were reconstituted as previously described (45).

##### PRDM9 Assay for Substrate Specificity Determination

A radioactivity-based assay was used to determine histone peptide substrate specificity of PRDM9. In this assay [^3^H]AdoMet (PerkinElmer Life Sciences; catalog number NET155V250UC) was used as a methyl donor to methylate biotinylated histone peptide substrates. Reaction mixtures contained 10 μm biotinylated peptide and 200 μm AdoMet (4 μm [^3^H]AdoMet plus 196 μm cold AdoMet) in 10 mm Tris-HCl buffer, pH 8.5, with 0.01% Triton X-100 and 10 mm DTT in a final volume of 20 μl. The reaction was started by adding PRDM9 (final concentration of 1 nm). Samples were incubated for 30 min at 23 °C and the reaction was quenched by addition of 20 μl of 7.5 m guanidinium hydrochloride followed by addition of 160 μl of 20 mm Tris-HCl buffer, pH 8.0. Samples were added to wells of a streptavidin/scintillant-coated microplate (FlashPlate® PLUS; PerkinElmer Life Sciences). Biotinylated peptides would be captured in each well through their interaction with streptavidin, thereby bringing any incorporated [^3^H]methyl in proximity of scintillant. The amount of methylated peptide was quantified by tracing the radioactivity (cpm) as measured by the TopCount NXT^TM^ Microplate Scintillation and Luminescence Counter (PerkinElmer Life Sciences).

##### Assay Optimization

The radioactivity-based assay described above was used to determine the best assay conditions for the PRDM9 methylation reaction. For the pH titration, the reaction mixture contained 10 μm biotinylated peptide and 40 μm AdoMet (2.5 μm [^3^H]AdoMet plus 37.5 μm cold AdoMet) in 20 mm Bis-tris propane buffer, pH 6.0–9.5, in a final volume of 20 μl. For the NaCl, DMSO, Triton X-100, DTT, and Tris(2-carboxyethyl)phosphine (TCEP) titrations, the reaction mixture contained 10 μm biotinylated peptide and 40 μm AdoMet (2.5 μm [^3^H]AdoMet plus 37.5 μm cold AdoMet) in 10 mm Tris-HCl, pH 8.5, in a final volume of 20 μl. The reactions were started by adding 100 nm PRDM9. Samples were incubated for 1 h at 23 °C and reactions were quenched by addition of 20 μl of 7.5 m guanidinium hydrochloride followed by addition of 160 μl of 20 mm Tris-HCl buffer, pH 8.0. Samples were added to wells of a streptavidin/scintillant-coated microplate (FlashPlate® PLUS: PerkinElmer Life Sciences). The amount of methylated peptide was quantified by tracing the radioactivity (cpm) as measured by a TopCount reader from PerkinElmer.

##### Determining Kinetic Parameters with H3-(1–25) and H3-(21–44) Peptides

A modified version of the radioactivity-based assay described above was used to measure the activity and kinetics of the PRDM9 reaction with H3-(1–25) peptide with un-, mono-, and dimethylated Lys-4 as well as H3-(21–44) with un-, mono-, and dimethylated Lys-36 as substrates. To avoid the binding capacity limitation of scintillation proximity assay plates, a membrane-based assay was used for all kinetic studies. Apparent *K_m_* values were determined for each set of peptides by varying the peptide concentration (0–9 μm and 0–20 μm for H3K4 and H3K36, respectively) and keeping the AdoMet at a saturation concentration of 448 μm (5 μm [^3^H]AdoMet plus 443 μm cold AdoMet). Apparent *K_m_* values were determined for AdoMet in reactions with each peptide by varying the AdoMet concentration. The highest concentration of AdoMet was 448 μm (5 μm [^3^H]AdoMet plus 443 μm cold AdoMet). Dilutions of AdoMet were then made with assay buffer. The peptide concentration was kept at 5 μm. The assay buffer was 10 mm Tris-HCl, pH 8.5, 0.01% Triton X-100, and 10 mm DTT in a final volume of 20 μl. The reactions were started by addition of 1 nm PRDM9. The reaction mixtures were incubated for 20 min at 23 °C and quenched by adding 20 μl of 7.5 m guanidinium hydrochloride. 10 μl of quenched reaction mixture was spotted onto streptavidin-coated membrane squares (SAM2® Biotin capture membrane, Promega). The membrane was washed three times in 2 m NaCl for 2 min each time, then in water three times for 30 s each. The membrane was dried at 50 °C for 1 h and then each spotted square was cut from the membrane and placed in a scintillation vial. The amount of methylated peptide was quantified by tracing the radioactivity (cpm) as counted by a TriCarb liquid scintillation counter (PerkinElmer Life Sciences).

##### Determining Kinetic Parameters with H3-H4 Tetramer

Transfer of methyl groups from [^3^H]AdoMet to the H3-H4 tetramer was monitored using the trichloroacetic acid (TCA) precipitation method in filter plates. The reactions (20 μl) were quenched by adding 180 μl of 10% TCA, followed by transferring the mixture to a 96-well filter plate (Millipore). The quenched reactions were filtered and washed twice each time with 100 μl of 10% TCA, and once with 100 μl of 100% ethanol, and allowed to dry, followed by addition of 70 μl of Microscint-O solution (PerkinElmer Life Sciences) to each well. The methylation was quantified by tracing the radioactivity (cpm) as measured by a TopCount reader from PerkinElmer.

##### Screening PRDM9 against a Known Histone Methyltransferase Inhibitor Library

The Z-factor was determined to ensure the optimized assay was amenable to high-throughput screening in a 384-well format (46). For this determination, 1 nm PRDM9 was screened with H3K36me2 peptide and AdoMet equal to their *K_m_* values (2.5 and 62 μm, respectively). PRDM9 was then screened against a small library of known histone methyltransferase inhibitors using H3K36me2 as substrate under optimized assay conditions. In this assay, 1 nm PRDM9 was incubated with 2.5 μm H3K36me2 peptide, 62 μm AdoMet in a reaction buffer (10 mm Tris-HCl, pH 8.5, 10 mm DTT, 0.01% Triton X-100) along with compounds ranging in concentrations from 0 to 100 μm, with a final DMSO concentration of 1%. Reactions were incubated at 23 °C for 30 min. The IC_50_ value was determined for suramin using SigmaPlot.

##### Mass Spectrometry Analysis

Mass spectrometry analysis was performed to confirm that PRDM9 is capable of methylation of un-, mono-, and dimethylated Lys-36 on the H3-(21–44) peptide. The reactions (40 μl final volume) contained 0.5 mm of each corresponding peptide, 1 μm PRDM9 and 1 mm AdoMet. The reactions were incubated at 24 °C for 5 h, quenched by addition of 110 μl of 0.5% formic acid, and immediately frozen on dry ice. For each reaction a blank was prepared by omitting the enzyme (PRDM9) from the reaction mixture. The samples were analyzed by an Agilent LC/MSD Time-of-Flight (TOF) mass spectrometer (Agilent Technologies, Santa Clara, CA) equipped with an electrospray ion source. To separate the peptide from the protein and remove buffer and salt, the samples were passed through a POROSHELL 300SB-C3 column using a 5–95% acetonitrile:water gradient.

##### Isothermal Titration Calorimetry (ITC)

Purified PRMD9 was dialyzed overnight in 20 mm Tris-HCl buffer, pH 8.5. Histone peptides or AdoMet at 1 mm in dialysis buffer were injected into the sample cell containing ∼1.4 ml of 30 or 50 μm PRDM9, respectively. ITC titrations were performed on a VP-ITC Micro Calorimeter from GE Healthcare at 25 °C by using 10-μl injections with a total of 25 injections. Data were fitted with a one-binding site model using Microcal Origin software.

##### Transfection and Immunoblotting of PRDM9 in Cells

A FLAG-tagged control vector or FLAG-tagged *PRDM9* construct were transfected into HEK293 cells using GeneJuice (Novagen). Transfected cells were serum-deprived prior to lysis in 50 mm Tris-HCl, 400 mm NaCl, 1 mm EDTA, 0.1% SDS, and 1% Triton X-100 supplemented with protease inhibitors. 30 μg of total cell lysate were processed for Western blotting and immunoblotted using specific antibodies to monitor the levels of H3K36me1 (Abcam, ab9048), H3K36me2 (Cell signaling Technology, 2901), H3K36me3 (Cell Signaling Technology, number 9763), H3K4me1 (Cell Signaling Technology, 9723), H3K4me2 (Abcam, ab32356), H3K4me3 (Cell Signaling Technology, number 9727S), and total H3 (Abcam, ab10799). The transfection efficiency of FLAG-PRDM9 was controlled by using an anti-FLAG antibody (FLAG M2 monoclonal, Sigma). Membrane bands were visualized using LiCor IRDye, 680RD anti-rabbit and 800CW anti-mouse secondary antibodies.

##### H3K36me2 Peptide Docking

α-Carbons of a GVKme2(36)KPH H3 peptide were tethered on the corresponding atoms of the RTKme2(4)QTA H3 peptide co-crystallized with PRDM9 (PDB code 4C1Q). The energy of the system was minimized by Monte Carlo simulation in the internal coordinate space, with fully flexible peptide and rigid PRDM9, using internal coordinate mechanics (47). Although the docking result looked satisfactory overall, H3P38 did not optimally exploit a neighboring hydrophobic cavity, probably to comply with conformational constraints imposed by the tethers. To generate the final model, the conformation of the three C-terminal residues was further optimized, in the absence of tether, with a second-step internal coordinate mechanics Monte Carlo simulation, where GVKme2(36) residues were kept rigid, and KPH flexible. Monte Carlo simulations were run twice independently and produced the same results, confirming in each case convergence toward an energy minimum.

## RESULTS

Although PRDM9 has been reported to trimethylate H3K4 *in vitro* and *in vivo* (28), its methyltransferase activity has not been fully characterized. To further investigate its substrate specificity and determine kinetic parameters for PRDM9 activity, we developed and optimized a radioactivity-based assay. In this assay the transfer of methyl groups from [^3^H]AdoMet to biotinylated peptide substrates was monitored using streptavidin-coated membranes and scintillation proximity assay plates. Preliminarily, we screened different soluble constructs of PRDM9 using H3-(1–25) peptide (first 25 residues of histone H3) and identified an active PR domain-containing construct corresponding to residues 195 to 385.

**FIGURE 1.**
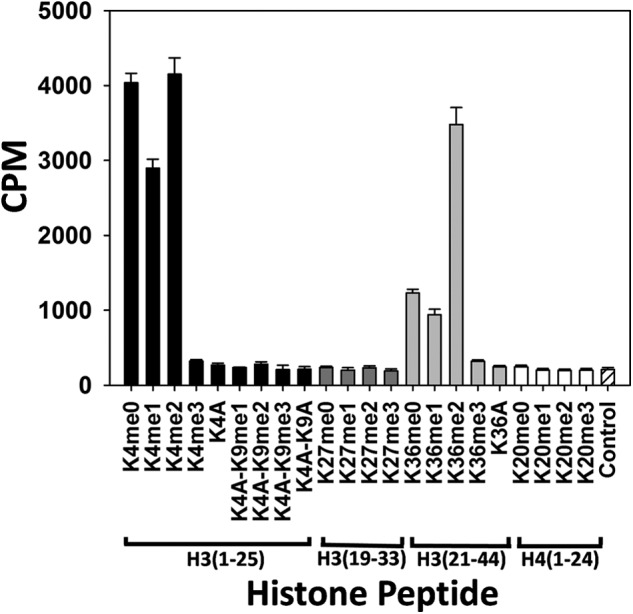
**PRDM9 substrate specificity.** Unmodified, lysine-mutated, and lysine-methylated H3-(1–25) peptides (*black bars*), H3-(19–33) peptides (*dark gray bars*), H3-(21–44) peptides (*light gray bars*), and H4(1–24) peptides (*white bars*) were tested for PRDM9 methyltransferase activity. A reaction containing the unmodified H3-(1–25) peptide, but no enzyme, was included as a control (*white bar* with *line pattern*). Data are presented as mean ± S.D. from four experiments.

**FIGURE 2.**
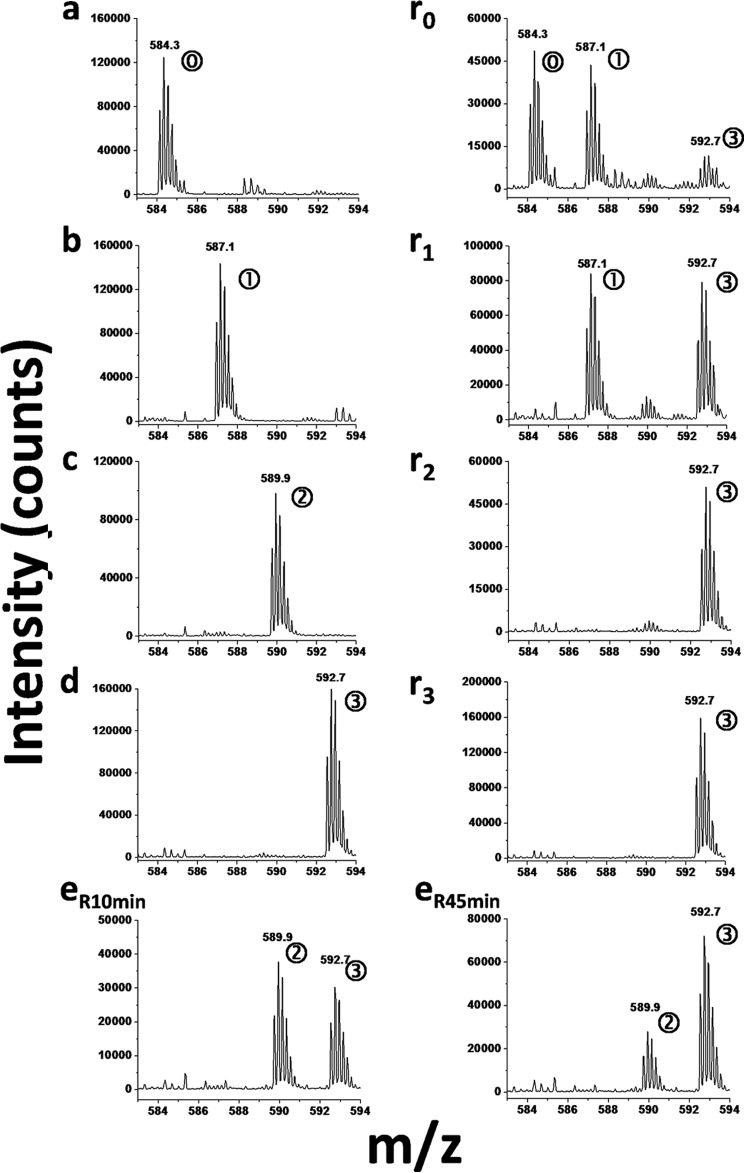
**Monitoring H3K36 methylation by PRDM9 using mass spectrometry.** The methylation state of H3K36 was monitored using LC-MS-TOF as described under “Experimental Procedures.” The mass spectra show the *m*/*z* signal for the +5-charged peptides corresponding to controls: unmethylated (H3K36me0; marked as “0” in a *circle* on the corresponding peak) (*a*); monomethylated (H3K36me1; 1) (*b*); dimethylated (H3K36me2; 2)(*c*); and trimethylated (H3K36me3; 3) (*d*) states. Spectra for reaction mixtures incubated for 5 h at 24 °C with unmethylated (*r*_0_), monomethylated (*r*_1_), dimethylated (*r*_2_), and trimethylated (*r*_3_) H3K36 peptide substrates confirm the methylation of H3K36 by PRDM9. The methylation state of each peptide is indicated by the *number of methyl groups in a circle* next to each peak. Methylation of H3K36me2 substrate after 10 (*e*_R10min_) and 45 min (*e*_R45min_) indicates the gradual reduction of dimethylated substrate and production of the trimethylated H3K36.

To determine the substrate specificity of PRDM9 a collection of peptides including two additional H3 peptides (H3-(19–33) and H3-(21–44)), as well as a peptide corresponding to the first 24 residues of histone H4 (H4(1–24)) were also tested (Fig. 1). In this screen, peptides with different methylation states of H3K4, H3K9, H3K27, H3K36, and H4K20 as well as K4A, K4AK9A, and K36A mutations were tested. PRDM9 was active with unmodified H3-(1–25) peptide, as well as with the mono- and dimethylated lysine 4 H3-(1–25) peptides (H3K4me0, H3K4me1, and H3K4me2, respectively). PRDM9 was not active with trimethylated H3K4 indicating that Lys-4 was the methylation mark for PRDM9 and the enzyme had no other methylation target on this peptide (Fig. 1). No activity was observed using K4A, K4AK9me1, K4AK9me2, K4AK9me3, and K4AK9A mutants of H3-(1–25) peptide as substrates, further confirming our conclusion that was consistent with previous reports (28). No activity was detected with the H3-(19–33) peptide with various Lys-27 methylation states. Similarly, PRDM9 was not active with the H4(1–24) peptide with various Lys-20 methylation states. Interestingly, PRDM9 was active with H3-(21–44) peptide with un-, mono-, and dimethylated Lys-36 as substrates, but not when Lys-36 was trimethylated or mutated to alanine (H3K36A) suggesting that Lys-36 may be a new methylation mark for PRDM9 (Fig. 1). We further confirmed the production of mono-, di-, and trimethylated H3K36 by PRDM9 using mass spectrometry (Fig. 2). Taken together, these results indicate the ability of PRDM9 to mono-, di-, and trimethylate residue H3K4 and H3K36 *in vitro*.

### 

#### 

##### Assay Optimization

**FIGURE 3.**
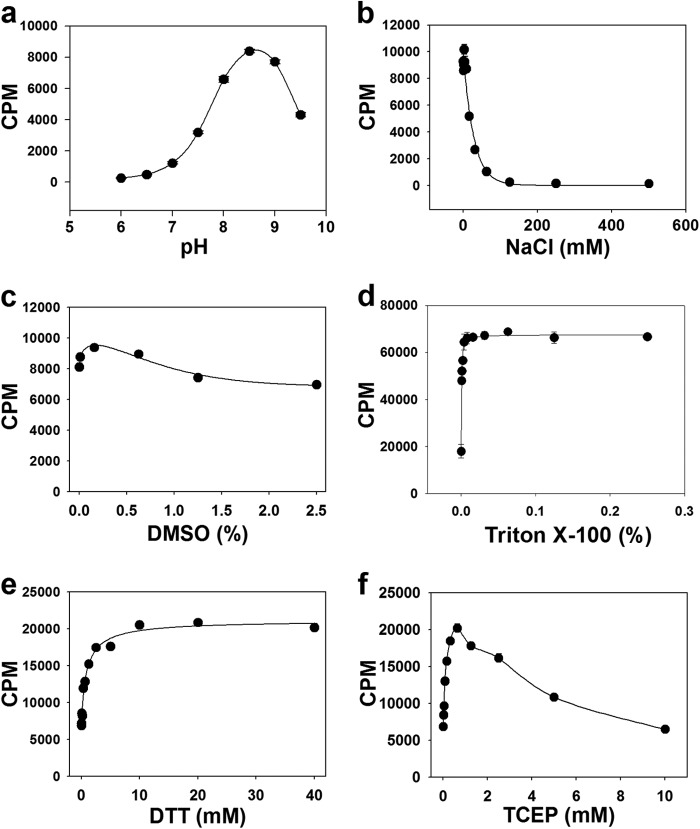
**Effect of pH, ionic strength, and buffer additives on PRDM9 activity.** The methyltransferase activity of PRDM9 using H3-(1–25) peptide as substrate was determined as a function of pH (*a*), NaCl (*b*), DMSO (*c*), Triton X-100 (*d*), DTT (*e*), and TCEP (*f*) as described under “Experimental Procedures.” Data points are presented as mean ± S.D. from four experiments.

PRDM9 activity was further characterized with respect to pH, salt (NaCl), reducing agents (DTT and TCEP) and additives (DMSO and Triton X-100). The enzyme showed maximum activity at pH 8.5 (Fig. 3*a*) in 20 mm Bis-tris propane buffer using H3-(1–25) as a substrate. Experiments were then repeated at pH 8.5 in various concentrations of Tris-HCl, Bis-tris propane, and Bicine buffers, and no significant difference was observed between different buffers (data not shown). All follow-up experiments were then performed using Tris-HCl buffer at pH 8.5.

The ionic strength of the buffer had a dramatic effect on PRDM9 activity with a sharp decrease in activity with increasing NaCl concentrations (Fig. 3*b*). Enzyme activity was abolished at NaCl concentrations above 125 mm. This negative effect of salt on enzyme activity might be due to interference with electrostatic interactions between the enzyme and its basic peptide substrate.

PRDM9 maintained high levels of activity at all concentrations of DMSO with maximum activity observed at 0.16% (Fig. 3*c*). The presence of Triton X-100 at concentrations as low as 0.004% dramatically improved the signal to noise ratio; Triton X-100 concentration was kept at 0.01% in all assays. Higher concentrations of Triton X-100 up to 0.25% did not further increase the activity (Fig. 3*d*).

The effect of reducing agents DTT and TCEP were also examined. Addition of DTT caused an increase in enzyme activity that reached the maximal effect at 10 mm, and was maintained at higher concentrations of DTT (Fig. 3*e*). Addition of TCEP on the other hand resulted in maximal enzyme activity at 0.6 mm, followed by a sharp decrease in activity at increasing TCEP concentrations (Fig. 3*f*). Considering these observations we subsequently used the following assay condition for all kinetic experiments: 10 mm Tris-HCl, pH 8.5, 0.01% Triton X-100, and 10 mm DTT.

##### Kinetic Parameter Determination

**FIGURE 4.**
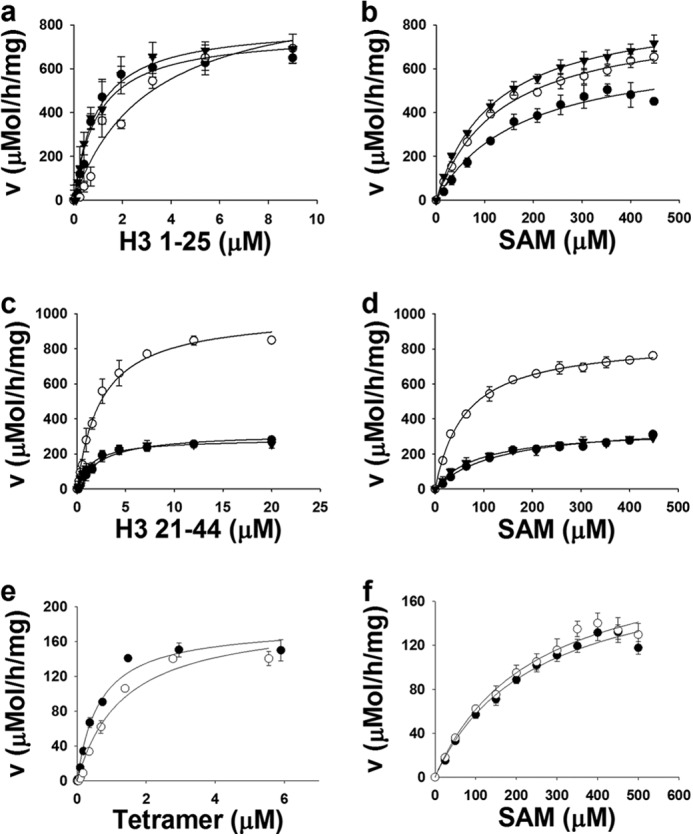
**Kinetic analysis of PRDM9 methyltransferase activity.** *K_m_* values were determined for H3-(1–25) peptides (H3K4me0 (▾), H3K4me1 (●), and H3K4me2 (○)) at saturation concentrations of AdoMet (*SAM*) (448 μm) (*a*) and for AdoMet with the same set of H3-(1–25) peptides (*b*). Similarly, *K_m_* values were determined for H3-(21–44) peptides (H3K36me0 (▾), H3K36me1 (●), and H3K36me2 (○)) at saturation concentrations of AdoMet (448 μm) (*c*) and for AdoMet with the same set of H3-(21–44) peptides (*d*). *K_m_* values were also determined for native (●) and truncated (○) H3-H4 tetramer in the presence of 350 μm AdoMet (*e*) and for AdoMet in the presence of 4.5 μm native (●) and 6.5 μm truncated H3-H4 tetramer (○) (*f*). In truncated H3-H4 tetramer, the first 11 amino acids of H3 were truncated. *V,* velocity. Data points are presented as mean ± S.D. from three experiments. Kinetic parameters are presented in Table 1.

Kinetic parameters were determined with H3-(1–25) peptide with un-, mono-, and dimethylated Lys-4 as well as H3-(21–44) with un-, mono-, and dimethylated Lys-36 as substrates as described under “Experimental Procedures” (Table 1). As expected, PRDM9 was inactive with trimethylated Lys-4 and Lys-36 peptides. The *K_m_* values for the unmodified H3-(1–25) peptide (H3K4me0) and H3K4me1 were the same (1 ± 0.2 μm; Table 1, Fig. 4*a*). The *K_m_* value for the H3K4me2 peptide was slightly higher (3 ± 1 μm). The *K_m_* values for AdoMet were also similar with H3K4me0, H3K4me1, and H3K4me2 peptides (120 ± 5 μm, 170 ± 30 μm and 140 ± 9 μm, respectively; Table 1 and Fig. 4*b*). Although catalytic efficiency (*k*_cat_/*K_m_*) of PRDM9 is slightly higher (∼3 times) for H3K4me0 than for H3K4me2, it efficiently mono-, di-, and trimethylates H3K4. With *k*_cat_ values as high as 19,000 ± 1,200 h^−1^ under these assay conditions, PRDM9 appears to be the most active histone methyltransferase known to date (Table 1) (48, 49).

**TABLE 1:** **Kinetic and binding parameters of PRDM9 and its substrates**

Substrate		*K*_*m*_^app^ *^a^*		*k*_cat_*^a^*	*k*_cat_/*K_m_*	*K_D_**^b^*
		Substrate	AdoMet			
		μ*m*		*h*^−*1*^	μ*m*^−*1*^ *h*^−*1*^	μ*m*
H3-(1–25)	K4me0	1 ± 0.2	120 ± 5	19,000 ± 1,200	19,000	3.6
	K4me1	1 ± 0.2	170 ± 30	15,000 ± 1,200	15,000	4.7
	K4me2	3 ± 1	140 ± 9	18,000 ± 900	6,000	5.8
	K4me3	NA*^c^*	NA	NA	NA	12
H3-(21–44)	K36me0	1.5 ± 0.2	87 ± 9	7,500 ± 400	5,000	4.6
	K36me1	2.4 ± 0.4	130 ± 32	5,000 ± 700	2,080	4.1
	K36me2	2.5 ± 0.3	62 ± 3	19,000 ± 500	7,600	4.2
	K36me3	NA	NA	NA	NA	9.6
H3-H4 tetramer	Native	0.7 ± 0.1	240 ± 40	4,100 ± 320	5,860	ND*^d^*
	Truncated H3	1.4 ± 0.3	240 ± 40	4,300 ± 300	3,070	ND

*^a^* Values are presented as mean ± S.D. from three experiments. Kinetic parameters are considered apparent values determined under the assay conditions described under “Experimental Procedures.”

*^b^* Values are presented as mean values from two experiments.

*^c^* NA, not active.

*^d^* ND, not determined.

**FIGURE 5.**
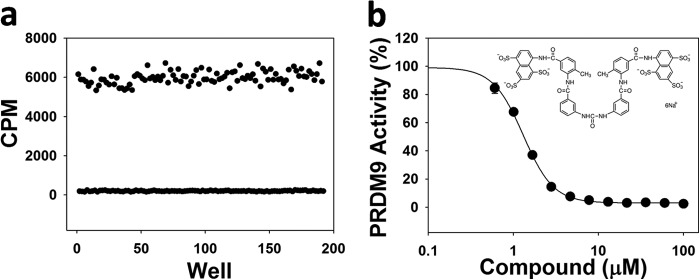
**Screening PRDM9 in 384-well format.** *a,* the Z-factor was determined (0.82) for PRDM9 screening with H3K36me2(21–44) peptide as a substrate at concentrations of peptide and AdoMet equal to their *K_m_* values (2.5 and 62 μm, respectively). *b,* PRDM9 was screened against a set of known histone methyltransferase inhibitors and suramin was identified as an inhibitor with an IC_50_ value of 4.1 ± 0.1 μm. Experiments were performed in triplicate as described under “Experimental Procedures.”

*K_m_* values for H3K36me0, H3K36me1, and H3K36me2 were also close (1.5 ± 0.2, 2.4 ± 0.4, and 2.5 ± 0.3 μm, respectively; Table 1 and Fig. 4*c*). The enzyme was highly active with H3K36me0, H3K36me1, and H3K36me2 peptides as substrate (*k*_cat_ of 7,500 ± 420 h^−1^, 5,000 ± 700 h^−1^, and 19,000 ± 500 h^−1^, respectively). The *k*_cat_ value for the reaction with H3K36me2 peptide is significantly higher than that of the H3K36me0 and H3K36me1 peptides. Dimethylated H3K36 appears to be a better substrate for PRDM9. The *K_m_* value for AdoMet was also determined in reactions with H3K36me0, H3K36me1, and H3K36me2 peptides as substrate (87 ± 9, 130 ± 32, and 62 ± 3 μm, respectively; Fig. 4*d*, Table 1). The optimized assay was amenable to screening in a 384-well format with a Z-factor (46) of 0.82 (Fig. 5*a*) and was used to screen a small library of known histone methyltransferase inhibitors. We found that suramin inhibits PRDM9 activity with an IC_50_ value of 4.1 ± 0.1 μm (Fig. 5*b*).

**FIGURE 6.**
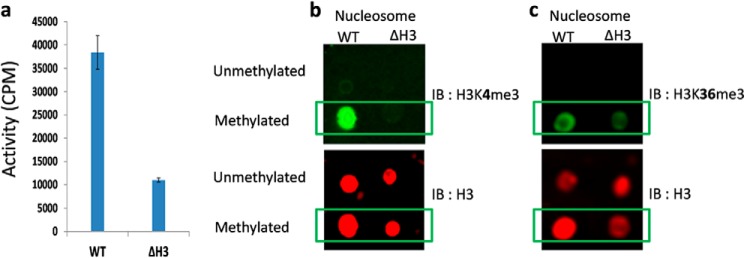
**H3K36 methylation on nucleosome.** Reconstituted nucleosome with recombinant wild-type histone H3 (*WT*) and truncated histone H3 (ΔH3; missing the first 11 residues including lysine 4) were used as substrate for PRDM9, and (*a*) the activity was assayed by monitoring the transfer of [^3^H]methyl groups from [^3^H]AdoMet to nucleosome using the trichloroacetic acid precipitation method as described under “Experimental Procedures.” Samples of each reaction mixture (*Methylated*) and untreated samples (*Unmethylated*; control) were immunoblotted using H3K4me3 (*b*) and H3K36me3 (*c*) antibodies (*upper panels* for *b* and *c*). The same dot blots were immunoblotted using histone H3 antibody (*IB*: H3) to quantify the amount of loaded nucleosome (*lower panels* for *b* and *c*).

Although we were able to detect the activity (∼16,000 cpm) of PRDM9 with human nucleosome (13.5 μm) extracted from HEK293 cells at a high concentration of enzyme (4 μm), we were not able to reach substrate saturation and the level of activity was too low for full kinetic studies. A low level of activity was also observed with recombinant nucleosome as substrate, making further studies on the nucleosome very challenging. However, high levels of activity were observed with the H3-H4 tetramer as a substrate (*k*_cat_: 4100 ± 320 h^−1^) (Table 1, Fig. 4, *e* and *f*). We also truncated the first 11 residues of histone H3 (eliminating H3K4) and reconstituted the H3-H4 tetramer. Truncated H3-H4 tetramer was also a good substrate for PRDM9 (*k*_cat_: 4,300 ± 300 h^−1^) despite deletion of H3K4, further supporting the H3K36 methylation (Table 1, Fig. 4, *e* and *f*). PRDM9 (4 μm) was also active (∼11,000 cpm) with nucleosomes reconstituted using truncated H3 (1.6 μm). Under the same conditions, PRDM9 was more active (∼38,000 cpm) with wild-type recombinant nucleosome (Fig. 6*a*). We further confirmed H3K36 methylation on truncated H3 nucleosome by immunoblotting using H3K36me3 antibody, and using H3K4me3 antibody as a control (Fig. 6, *b* and *c*). Altogether, our data clearly confirms H3K36 methylation by PRDM9.

##### Substrate Binding Studies

**FIGURE 7.**
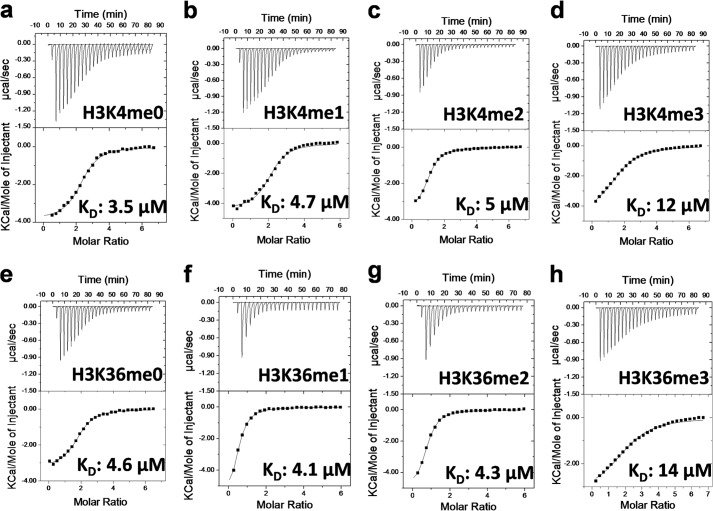
**Assessing interactions of PRDM9 with substrates.** Binding of H3-(1–25) and H3-(21–44) peptides with various H3K4 and H3K36 methylation states was monitored by ITC as described under “Experimental Procedures.” Peptide substrates and their corresponding *K_D_* values from a single run are indicated on the plots. Experiments were performed in duplicate. Mean *K_D_* values are listed in Table 1.

Binding affinities of peptide substrates and AdoMet were determined using ITC as described under “Experimental Procedures” (Fig. 7). Consistent with an observed decrease in activity in the presence of high salt (Fig. 3*b*), very weak peptide binding was observed when performing the ITC experiments in buffers with NaCl such as PBS buffer. Using 20 mm Tris-HCl pH 8.5 buffer, which was similar to the enzyme assay conditions, appeared to be the optimum buffer condition for ITC experiments as well. The difference in *K_D_* values for un-, mono-, and dimethylated Lys-4 and Lys-36 peptides were not significant (ranging from 3.6 to 5.8 μm). However, *K_D_* values for H3K4me3 and H3K36me3 peptides (12 and 9.6 μm, respectively) were significantly higher (Fig. 7, Table 1). The *K_D_* value for AdoMet was 18.6 μm.

##### Cell-based Assays

**FIGURE 8.**
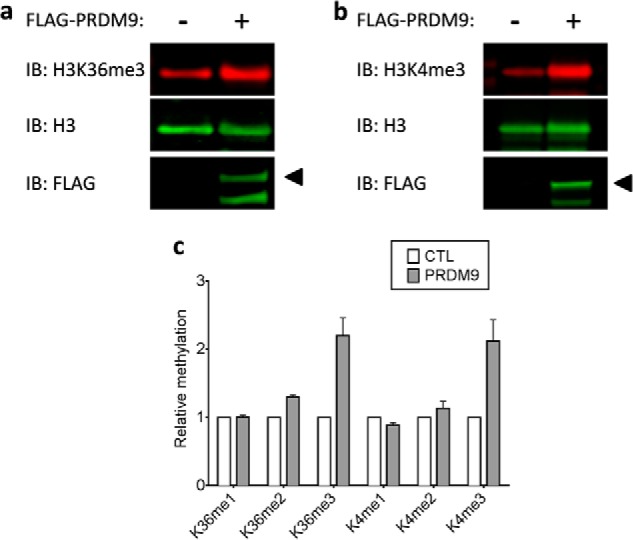
**Trimethylation of H3K4 and H3K36 by PRDM9 in cells.** Trimethylation of H3K36 (*a*) and H3K4 (*b*) in HEK293 cells transfected with control and PRDM9 plasmids was monitored by Western immunoblotting. *c*, blot quantifications indicate significant H3K4 and H3K36 trimethylation in samples from cells overexpressing PRDM9. Methylation levels of Lys-4 and Lys-36 were quantified and normalized to their respective total H3 levels. FLAG-PRDM9-transfected samples were normalized to samples transfected with FLAG Control vector. *IB*, immunoblot; *CTL*, control.

To investigate the H3K4 and H3K36 methylation activity of PRDM9 in cells, a FLAG-tagged PRDM9 plasmid was transfected into HEK293 cells. H3K4 and H3K36 levels of methylation were monitored by immunoblotting using specific antibodies for methylation states of each mark as specified under “Experimental Procedures” (Fig. 8). The immunoblot quantifications showed a significant increase in trimethylation of H3K4 and H3K36 in cells overexpressing PRDM9, but not mono- or dimethylation of either mark (Fig. 8*c*). The cell-based data indicate that PRDM9 is capable of trimethylating H3K36 in HEK293 cells.

##### H3K36me2 Peptide Docking

**FIGURE 9.**
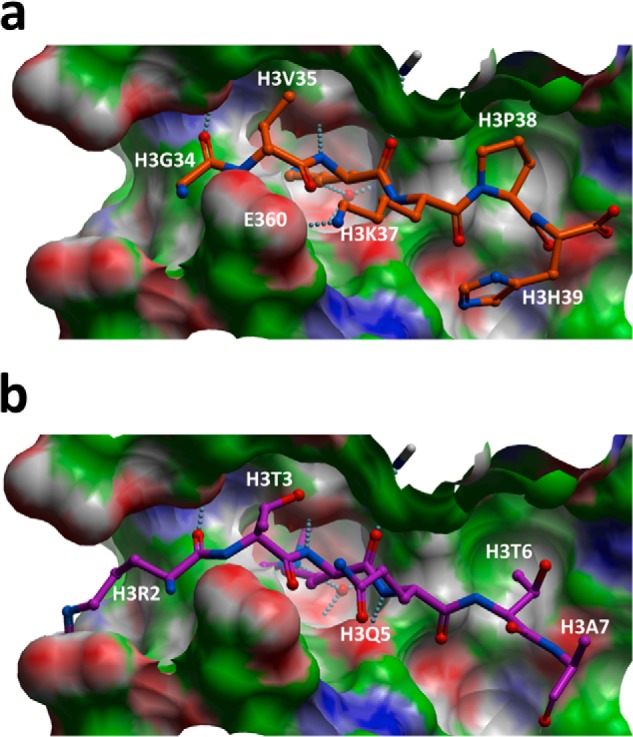
**H3K36me2 peptide docking.** The docked H3K36me2 peptide (*a*) recapitulates interactions observed with the backbone of the co-crystallized H3K4me2 peptide (*b*). H3V35 and H3P38 side chains make favorable desolvation contacts with hydrophobic clefts of the substrate-binding groove of PRDM9, and H3K37 is engaged in an electrostatic interaction with Glu-360.

To better understand the structural determinants for H3K4/H3K36 dual specificity, we docked the H3K36me2 peptide GVKme2(36)KPH to PRDM9 (Fig. 9). Hydrogen bonds engaged with the backbone of the histone peptide observed in the PRDM9-H3K4me2 crystal structure (PDB code 4C1Q) were conserved in the H3K36me2 complex. H3V35 and H3P38 side chains favorably exploit hydrophobic cavities occupied by H3T3 and H3T6, respectively, in the H3K4me2 complex. Interactions formed with the aliphatic side chain of H3R2 are lost, but H3K37 forms an additional electrostatic interaction with Glu-360. In summary, the model reveals how the substrate-binding groove of PRDM9 has acquired structural features that can accommodate two distinct histone sites.

## DISCUSSION

PRDM9 is a PR domain-containing protein with multiple zinc fingers that catalyze trimethylation of H3K4 (28). It is a meiosis-specific protein that has been reported to control activation of the short highly recombinogenic segments called recombination hot spots (50). The H3K4me3 mark is often associated with transcriptional activation (51, 52) and DNA repair (53). This methyl mark is also associated with recombination hot spot initiation in yeast and mice (35, 36) where double strand breaks occur prior to crossover events. However, H3K4me3 marks associated with hot spots are distinct from those at transcription start sites (37). It was reported that 87% of double strand break hot spots in mice overlap with testis-specific H3K4me3 marks (37). PRDM9 binds *in vitro* to a 13-mer DNA sequence enriched in human hot spots (32, 33) where it initiates recombination by its H3K4 trimethylation activity. Disruption of PRDM9 expression in mice caused sterility (28) and addition of PRDM9 via baculovirus to sterile mouse hybrids rescued the sterile phenotype (54). In human, a handful of *PRDM9* SNPs have been shown to be associated with azoospermia and sterility as well (40, 41). The expression of PRDM9 in male germ cells and its role in the progression of meiosis make PRDM9 a candidate target for pharmacologic compounds that may work as non-hormonal, reversible male contraceptives. Recently, *PRDM9* variability in humans has been implicated in genome instability and having a potential role in the risk of acquiring genome rearrangements associated with childhood leukemogenesis (44). PRDM9 has a role in genetic diversity and proper progression of meiosis, and a possible increase in risk for a variety of diseases upon change in its activity (31, 33, 44). This raises the question of whether or not variability of PRDM9 and change in its level of activity may contribute to an increased risk for other rearrangement-associated diseases.

Although PRDM9 H3K4 methyltransferase activity has been previously reported (28), to our knowledge its activity and substrate specificity have not been fully characterized. Our kinetic characterization of the PRDM9 H3K4 methyltransferase activity revealed that PRDM9 is one of the most active histone methyltransferases with *k*_cat_ values of 19,000, 15,000, and 18,000 h^−1^ with un-, mono-, and dimethylated H3K4 substrates, respectively. Our binding data also indicated tight H3K4 peptide binding (low micromolar). Previously, Hayashi *et al.* (28) reported that GST-PRDM9 specifically catalyzed methylation of native calf thymus histone H3 but not recombinant GST-fused full-length H3, even when the GST tag was removed from the substrate. They also observed only trimethylation when peptides were used as substrates and in cells. Very recently PRDM9 was reported to also mono- and dimethylate H3K4 in addition to catalyzing the H3K4 trimethylation (55). Using un-, mono-, and dimethylated peptides as substrate, we did observe, quantify, and characterize the ability of PRDM9 to mono-, di-, and trimethylate H3K4 and H3K36, a novel mark for PRDM9. Binding of PRDM9 to a histone H3-(26–45) peptide on a peptide array was also recently reported that implicated H3K36 as a possible methylation site (55). To further confirm the H3K4/H3K36 monomethylation by PRDM9, we used recombinantly reconstituted H3-H4 tetramer as substrate. PRDM9 indeed methylated H3-H4 tetramer indicating that PRDM9 is capable of catalyzing the monomethylation of its substrates. As PRDM9 di- and trimethylates the monomethylated product very efficiently, it would not be unexpected that the marks would mainly be found in the trimethylated state in cells as previously reported (28) and observed in HEK293 cells.

When screening for other possible PRDM9 substrates, we also discovered H3K36 as a novel target residue for PRDM9 methylation using peptide substrates. Methylation of the H3-H4 tetramer and nucleosome when H3 was N terminally truncated further supported H3K36 methylation by PRDM9. H3K36 methylation on truncated nucleosome was further confirmed by immunoblotting. Interestingly, it catalyzed mono-, di-, and trimethylation of H3K36 *in vitro* with levels comparable with H3K4 methylation. Importantly, we showed that overexpression of PRDM9 in HEK293 cells resulted in a significant increase in H3K36 trimethylation as well as H3K4 trimethylation confirming our *in vitro* data.

The H3K36me3 mark is found on expressed exons, *versus* introns, which supports its involvement with transcriptional elongation and splicing (56). Additionally, H3K36me3 regulates DNA mismatch repair through its recruitment of the PWWP domain of MSH6 (57). SETD2, a protein that behaves like a tumor suppressor gene in breast cancer (58) and clear cell renal cell carcinoma (59), also trimethylates H3K36 (13). SETD2 transcripts are found in several human tissues, including testis (60), and the gene was also found to be significantly expressed in the testis compared with 10 other human tissues (61). Although we observed an increase in trimethylation of H3K36 upon overexpression of PRDM9 in HEK293 cells, further studies are needed to show PRDM9-dependent H3K36 trimethylation during meiosis and to clarify its effect and physiological relevance.

## CONCLUSION

We have found that PRDM9 is able to trimethylate H3K36 *in vitro* and in HEK293 cells. The physiological significance of this reaction is not yet known. We also found that PRDM9 was able to mono- and dimethylate H3K4 *in vitro*, and we confirmed earlier reports of its ability to trimethylate H3K4 *in vitro* and in HEK293 cells. The restrictive expression of PRDM9 in testis and its association with sterility make this protein a desirable target for male contraceptive design. Compound screens will isolate potential PRDM9 inhibitors to this end.

## Footnotes

4 The abbreviations used are:

AdoMet: *S*-adenosylmethionine
H3K4: histone H3 lysine 4
PRDM: PR domain-containing protein
TCEP: Tris(2-carboxyethyl)phosphine
ITC: isothermal titration calorimetry
Bis-tris propane: 1,3-bis[tris(hydroxymethyl)methylamino]propane
HMT: histone methyltransferase
NSD: nuclear receptor SET domain
Bicine: *N*,*N*-bis(2-hydroxyethyl)glycine
DMSO: dimethyl sulfoxide.
